# Supplementary figures and images for: Genomic and phenotypic diversity of Enterococcus faecalis isolated from endophthalmitis
Source: PLoS One. 2021 Apr 14;16(4):e0250084. doi: 10.1371/journal.pone.0250084 (PMC8046195; doi:10.1371/journal.pone.0250084)

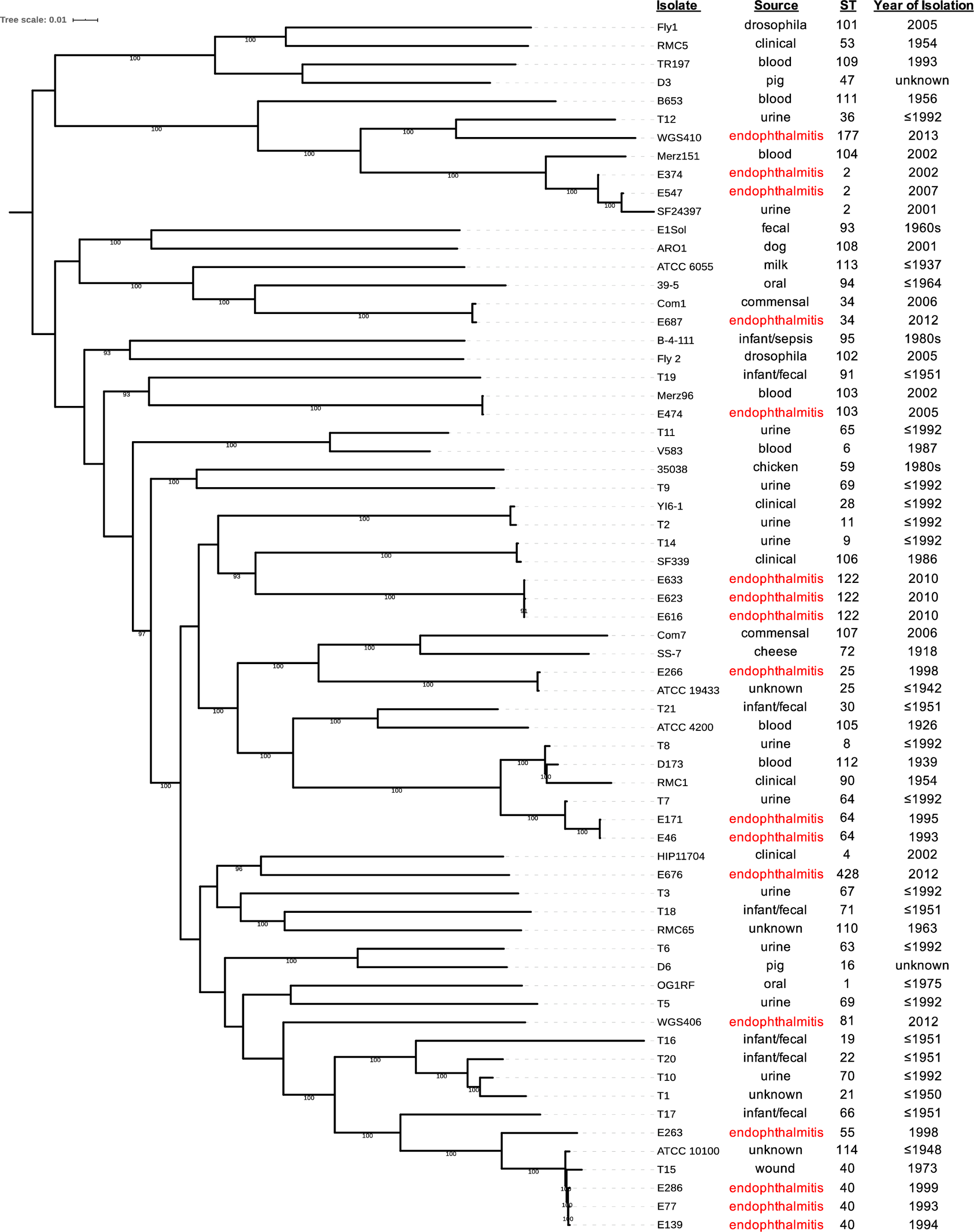

Supplement: S1 Fig — A core genome alignment was generated for 1,710 core genes with Roary, and the phylogeny was made with RAxML. Tips are labeled with isolate name, source, sequence type (ST), and year of isolation. Isolates from endophthalmitis are labeled red. Bootstrap values >90 are shown on the tree. (TIF) [file pone.0250084.s001.tif]

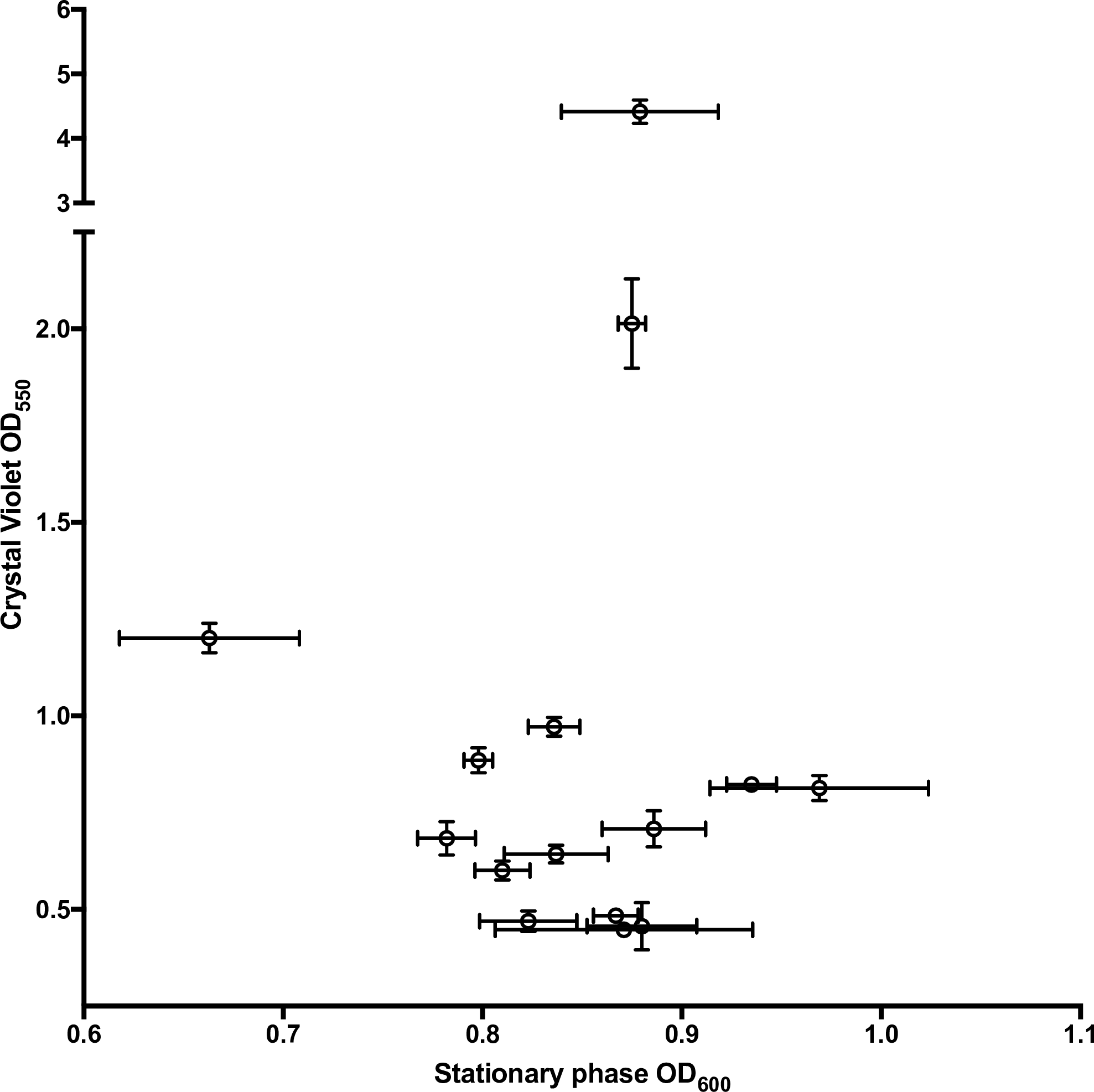

Supplement: S2 Fig — Crystal violet OD550 values from a standard in vitro biofilm assay were compared with stationary phase OD600 values for the same isolates grown overnight in Brain Heart Infusion media supplemented with 0.25% glucose. The mean value for each isolate is plotted, and error bars indicate the standard error of the mean of 24 measurements for biofilm staining and 9 measurements for stationary phase cell density. (TIF) [file pone.0250084.s002.tif]

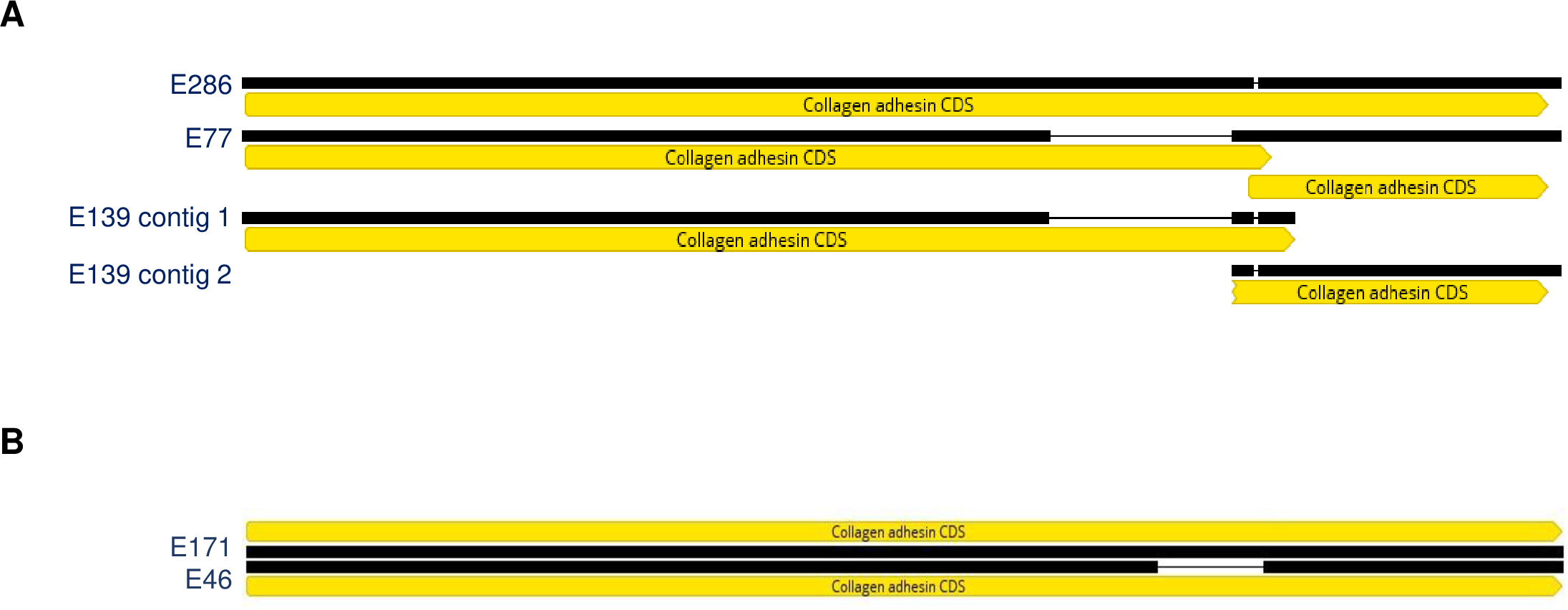

Supplement: S3 Fig — Nucleotide sequence alignment of the ace sequences from (A) ST40: E286, E77 and E139, and (B) ST64: E171 and E46 isolates. Yellow arrows show the ace coding sequence, black bars show nucleotide regions that align with one another, and black lines show deletions in isolates that form less biofilm. (TIF) [file pone.0250084.s003.tif]
