# Supplementary material for: Genomic and phenotypic diversity of Enterococcus faecalis isolated from endophthalmitis
Source: PLoS One. 2021 Apr 14;16(4):e0250084. doi: 10.1371/journal.pone.0250084 (PMC8046195; doi:10.1371/journal.pone.0250084)
Supplement: Attachment — Submitted filename: Response to reviewers_feb27.docx [file pone.0250084.s011.docx]

We thank the editor and reviewers for their insightful comments, and have revised our manuscript based on their feedback. These changes are summarized below, and revisions to the manuscript text are highlighted in yellow in the marked-up manuscript pdf file.

Reviewer #1:

This paper analyzed the genetic variation and phenotypic character of Enterococcus faecalis, which was identified as the causative agent of endophthalmitis, and reported its diversity. A total of 17 strains were used for the analysis, of which 15 were used at the center to which the author belongs, and 2 were reported. Whole genome sequences were analyzed and antibiotic susceptibility tests, biofilm and cytolysin assays, and genetic mutation assays were conducted to compare gene changes and phenotypic characteristics. Several experiments have shown quite good results, but the number of individuals is small, so it seems that it is not possible to draw a definite conclusion. However, infectious endophthalmitis occurs not only in one in tens of thousands of people, but also *E. faecalis* is less likely to occur as a causative agent. Recognizing these limitations, this paper can be seen as helpful in providing motives for various researches.

- We thank the reviewer for these comments. We agree that even though *E. faecalis* endophthalmitis is an extremely rare condition, it nonetheless warrants further characterization.

1. For the antibiotic resistance of fluoroquinolones, you have checked the mutation of gyrA. For parC, please describe how the mutation is. In fact, the reality is that there are few antibiotics available in eye clinic for infection control. Therefore, you may be more interested in antibiotic resistance to the first to fourth generations of fluoroquinolones, vancomycin, ceftazidime, cefuroxime, than other antibiotics. I hope that further research will be done on this.

- We thank the reviewer for bringing this to our attention. We checked the *parC* sequences in our isolates and identified S82I mutations in the fluoroquinolone-resistant isolates. This finding is now included in the revised manuscript (Lines 278-282, Line 392, and Table 2). We agree with the reviewer that further research is needed in this area.

2. E. faecalis, which is derived from endophthalmitis, can be very rare, but is relatively common in other diseases. Regarding this background, I think that comparing E. faecalis cultivated in other diseases can supplement the limitations of the paper.

- We agree with the reviewer that our single center study is limited to a small sample set; nonetheless, our study is the largest to date focused on comparative genomics and phenotypic characterization of *E. faecalis* from endophthalmitis. In Figure S1 we compare the phylogenetic relationship of the isolates we collected from post-operative endophthalmitis with publicly available genomes of commensal, clinical and environmental *E. faecalis* from NCBI. We observed that *E. faecalis* derived from endophthalmitis are not restricted to a particular lineage, but instead resembled *E. faecalis* collected from both clinical and non-clinical sources. Numerous other studies have described *E. faecalis* collected from other diseases; here our goal was to highlight the genomic and phenotypic diversity of *E. faecalis* from post-operative endophthalmitis. We hope to conduct larger, multi-center studies with larger sample sets in the future.

3. Recently, there are papers that E. faecalis is increasing as a causative isolate of postoperative endophthalmitis. There are reports of these in Sweden, Taiwan, and Korea, and it would be nice to add them to the introduction.

- Thank you for this feedback. We have now included these reports in the Introduction (Lines 53-55).

Reviewer #2: 
This is an interesting, well-written, well-presented study of enterococcus samples that caused endophthalmitis at single institution. Known E. faecalis virulence factors (cytolysin and biofilm production) and antibiotic resistance genes in each genome were analyzed. Interestingly, the authors found evidence of E. faecalis adaptation during recurrent endophthalmitis by identifying genetic variants that arose in sequential isolates sampled at different time points from the same patient, however, it is not clear if the mutation arose during the recurrent endophthalmitis within the patient or if it was selected during the extended period of incubation after isolation. All of the samples were susceptible to vancomycin.

- We are happy to learn that the reviewer found our study to be interesting and well written.

While the variability in the virulence factors is interesting, it would be especially interesting to see if there was any clinical correlation between these virulence factors and outcomes.

- We thank the reviewer for their feedback. At the time of isolate collection, all bacterial isolates were deidentified, and unfortunately no metadata associated with the patients who provided these isolates were retained. Many of these patients were referred to the UPMC Eye Center from outside facilities, and some of the isolates were collected nearly three decades ago. While we would have liked to be able to draw connections between the virulence factors we identified and clinical outcomes, it simply is not possible given how the isolates were collected. We have noted this in the revised manuscript (Lines 368-369).
